# Temporal Network Analysis of Bedtime Procrastination and Depression Among Adolescents: A Prospective Longitudinal Study

**DOI:** 10.1002/mco2.70620

**Published:** 2026-01-25

**Authors:** Tingting Gao, Wei Zhang, Yingying Su

**Affiliations:** ^1^ Department of Social Medicine and Health Management School of Public Health Cheeloo College of Medicine Shandong University Shandong China; ^2^ NHC Key Lab of Health Economics and Policy Research (Shandong University) Shandong China; ^3^ Center For Health Management and Policy Research Shandong University (Shandong Provincial Key New Think Tank) Shandong China; ^4^ School of Public Health Wannan Medical College Wuhu China; ^5^ School of Public Health and Emergency Management Southern University of Science and Technology Shenzhen China

**Keywords:** adolescents, bedtime procrastination, depression, longitudinal study, network analysis

## Abstract

Research on bedtime procrastination and depression has primarily used traditional psychometric approaches, limiting the ability to capture symptom‐level temporal dynamics. This study aimed to examine within‐ and between‐person associations between symptoms of depression and bedtime procrastination, considering sex differences. Data from 3296 adolescents followed over 18 months were used to explore symptom associations and their centrality in cross‐sectional networks, as well as cross‐lagged effects to clarify temporal relationships. In the within‐person temporal network, *not going to bed on time* and *trouble concentrating* were the most influential symptoms for males and females, respectively. In the contemporaneous network, *feeling worried* and *not going to bed on time* were most central for males, while *feeling tired* and *feeling worried* were central for females. No significant sex differences were found in overall network strength (*S* = 0.10, *p* = 0.75) or structure (*M* = 0.48, *p* = 0.09). Positive associations were consistent at the between‐person level. Overall, this study characterizes the symptom‐to‐symptom associations between depression and bedtime procrastination at both the within‐ and between‐person levels, with notable sex differences. For males, sleep difficulties and worries were key factors, while for females, concentration issues and fatigue played a more significant role.

## Graphical abstract

This study explored symptom‐to‐symptom associations between bedtime procrastination and depression among 3296 adolescents over 18 months. Temporal network analyses revealed that “not going to bed on time” and “trouble concentrating” were key symptoms for males and females, respectively. Findings highlight dynamic within‐person links and provide a foundation for personalized mental health interventions.

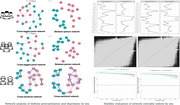

## 1 Introduction

During adolescence, delayed sleep patterns are common, often resulting in reduced sleep duration on school nights. Asian adolescents in particular exhibit later bedtimes compared to peers in North America and Europe [1]. One behavioral pattern that may contribute to these delayed sleep schedules is bedtime procrastination. It refers to the voluntary delay of going to bed at the intended time, even when one has sufficient opportunity and no external constraints preventing sleep [2, 3]. In addition to causing sleep deprivation, the effects of bedtime procrastination are also significant for poor sleep quality [4], increased daytime fatigue [5], and the development of mental health issues [6]. Depression is also a prevalent mental health disorder for adolescents and is projected to become one of the leading causes of disease burden worldwide by 2030 [7]. From 2001 to 2020, the global prevalence of self‐reported depression reached 34% in adolescents aged 10–19 years, with higher prevalence observed in Asia [8]. Adolescent depression has been linked to various negative outcomes, including, but not limited to, failure to complete school, internet addiction [9], adult anxiety [10], and suicidal behavior [11].

Empirical studies showed that bedtime procrastination predicted subsequent depressive symptoms [12, 13]. Conversely, as a form of negative affect, depression can significantly impact bedtime procrastination, and this relationship can be elucidated through the framework of the Self‐Regulation Resource Model (SRRM) [6, 14, 15]. According to the model, depression redirects limited self‐regulatory resources toward immediate mood repair, consuming resources that would otherwise be allocated to long‐term goals, such as maintaining a healthy sleep schedule [16]. Additionally, depression may narrow an individual's temporal perspective, promoting short‐term gratification behaviors such as staying up late [17]. Therefore, bedtime procrastination represents a maladaptive emotion regulation strategy, wherein individuals delay sleep to cope with immediate depressive moods [18]. Although a reciprocal longitudinal relationship between bedtime procrastination and depression may exist, this association requires further empirical validation beyond traditional latent variable approaches.

Network analysis provides a comprehensive framework for examining how individual symptoms interact dynamically over time, moving beyond the assumption of an underlying latent disorder that gives rise to observable symptoms [19]. This approach conceptualizes psychopathology as an emergent system in which disorders are represented as networks. Each symptom functions as a node, and edges represent direct associations or causal relationships, forming self‐sustaining feedback loops that may lead to the onset and maintenance of disorders [20]. By quantifying direct associations between symptoms, network analysis enables the identification of central symptoms, those most strongly connected to others, which are considered potential targets for early intervention and treatment optimization [21, 22, 23, 24]. Research has applied network models to investigate the interplay between depression and sleep‐related symptoms, revealing that certain symptoms (e.g., difficulty initiating sleep, fatigue, and depressed mood) consistently occupy central positions within these networks [21]. Nevertheless, these studies rely on cross‐sectional designs, which capture associations between symptoms at a single time point but cannot clarify their temporal order or the directional and causal structure of symptom changes over time. In contrast, longitudinal network models enable us to examine how these relationships evolve dynamically, providing more robust insights into potential causal pathways. Despite increasing interest in this approach, there remains a notable gap in longitudinal research investigating item‐level dynamic networks connecting bedtime procrastination and depression among adolescents. To address this, the cross‐lagged panel network (CLPN) model has recently been proposed as an advanced analytic framework that integrates traditional cross‐lagged panel modeling with network analysis [25]. This approach allows for the simultaneous estimation of autoregressive and cross‐lagged pathways among symptoms over time, capturing both symptom stability and directional influences. Recent empirical work has demonstrated the utility of this framework; for example, Chen et al. applied CLPN to examine longitudinal depressive symptom dynamics within father‐mother‐child triads, showing how specific symptoms exert directional influence across individuals over time [26]. Importantly, researchers have emphasized the significance of differentiating between within‐person and between‐person associations [27, 28]. Within‐person effects reflect intraindividual fluctuations, where an increase in one symptom predicts a subsequent rise in another symptom within the same individual over time. In contrast, between‐person effects capture interindividual differences, suggesting that individuals with consistently higher levels of one symptom are also likely to experience higher levels of other symptoms [29].

Notably, the dynamics of depression and bedtime procrastination are likely to exhibit distinct patterns depending on sex, as studies in adolescents have revealed significant sex differences in symptom structure [30]. Similarly, research has identified sex‐specific variations in bedtime procrastination behavior, with differences observed both between depressed and non‐depressed individuals and across varying levels of depression severity [14]. However, these findings are largely based on cross‐sectional studies, and it remains unclear whether similar sex‐specific patterns manifest in longitudinal, item‐level networks connecting bedtime procrastination and depressive symptoms, distinguishing between within‐person and between‐person effects. To address this gap, the present study aimed to examine the longitudinal, item‐level relationships between bedtime procrastination and depressive symptoms in adolescents over an 18‐month period using CLPN modeling. Specifically, we aimed to explore how individual symptoms of bedtime procrastination and depression influence each other over time, distinguishing both within‐person and between‐person effects, and examining potential sex‐specific patterns. We also aimed to identify key symptoms that may serve as potential targets for intervention in adolescent populations.

## 2 Results

**FIGURE 1:**
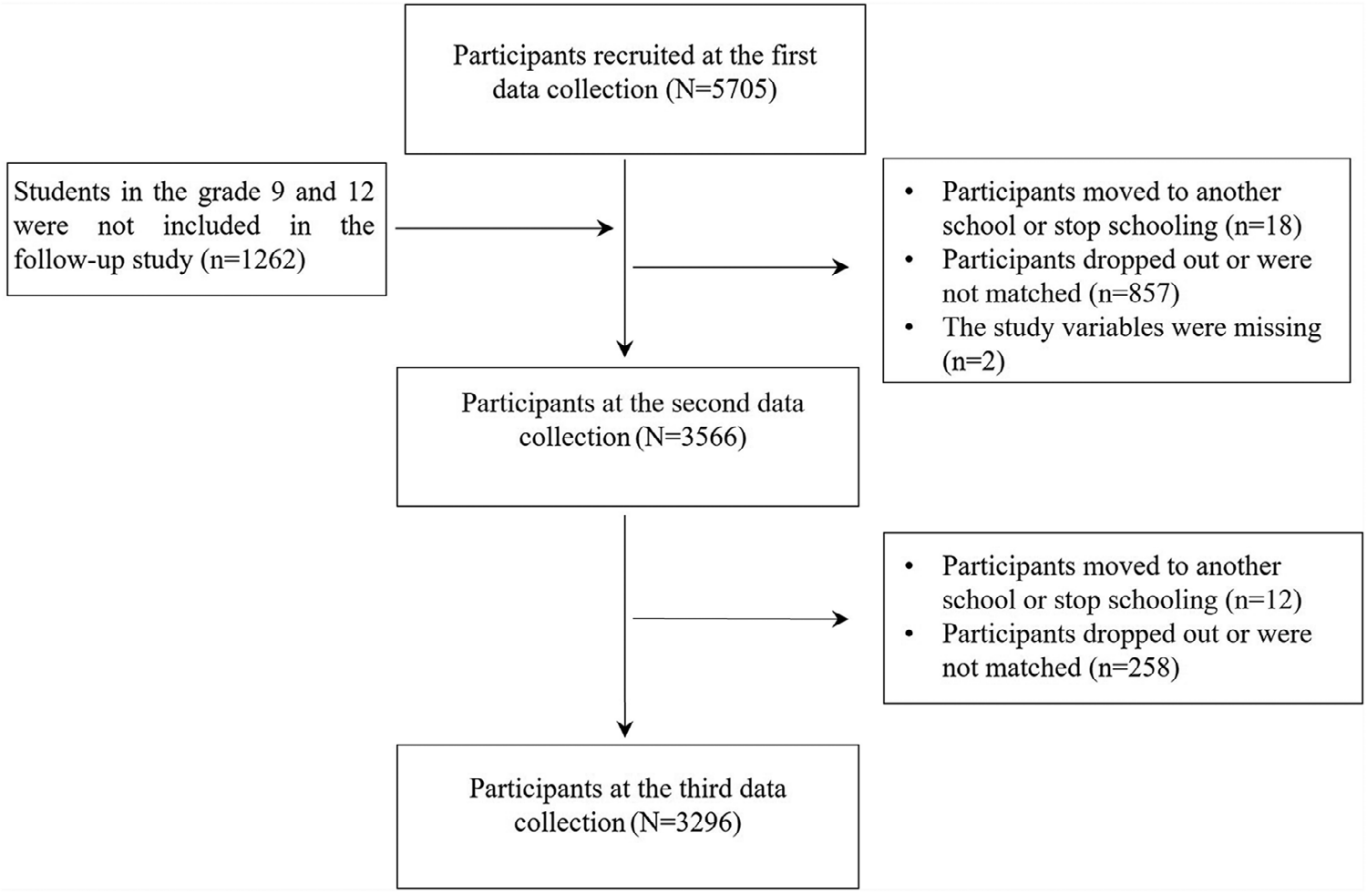
Flow chart of participants included in the analysis, illustrating the participant selection process.

A total of 3296 students (1795 females; 2501 from rural areas) were included in this study. In the first survey, students aged 12–19 years had a mean age of 15.17 ± 1.44 years. The study sample comprises various grade levels as follows: 37.7% (*n* = 1243) in Grade 11, 35.4% (*n* = 1166) in Grade 10, 13.5% (*n* = 445) in Grade 8, and 13.4% (*n* = 442) in Grade 7. A detailed description of the sampling procedure used to select study participants is depicted in Figure 1. Additionally, we compared the demographic characteristics of the non‐participating and participating groups. The non‐participating and participating groups showed no significant differences in age (*t* = 1.83, *p* = 0.07), sex (*χ* ^2^ = 0.45, *p* = 0.50), or whether they came from a one‐child family (*χ* ^2^ = 0.49, *p* = 0.49). Details of all items, including their exact wording, abbreviations, and conceptual domains, are also provided in Table 1.

**TABLE 1:** Items of the Kutcher Adolescent Depression Scale (KADS) and Bedtime Procrastination Scale (BPS).

Scale	Exact item wording	Abbreviation	Conceptual domain
Kutcher Adolescent Depression Scale (KADS)	Physical feelings of worry	KADS1	Anxiety/physiological arousal
	Thoughts, plans, or actions about suicide or self‐harm	KADS2	Suicidality
	Feelings of worthlessness, hopelessness, letting people down, and not being a good person	KADS3	Self‐esteem/cognitive‐affective
	Feeling worried, nervous, panicky, tense, keyed up, anxious	KADS4	Anxiety/affective tension
	Irritable, losing your temper easily, feeling pissed off, losing it	KADS5	Irritability/affective dysregulation
	Trouble concentrating	KADS6	Cognitive impairment/attention
	Feeling that life is not very much fun	KADS7	Anhedonia/loss of interest
	Low mood, sadness, feeling blah or down, depressed, just can't be bothered	KADS8	Depressed mood
	Feeling tired, feeling fatigued, low in energy, hard to get motivated, have to push to get things done, want to rest or lie down a lot	KADS9	Fatigue/somatic energy
	Feeling decreased interest	KADS10	Anhedonia/motivation
	Sleep difficulties	KADS11	Sleep disturbance/somatic
Bedtime Procrastination Scale (BPS)	I go to bed later than I had intended	BPS1	Intention–behavior gap/behavioral delay
	Even if I don't have to get up early the next day, I still go to bed early	BPS2	Routine adherence/self‐regulation
	When it is time to turn off the lights and sleep, I do so immediately	BPS3	Sleep hygiene/behavioral control
	I find myself still doing other things when it is time to go to bed	BPS4	Distraction/behavioral delay
	Even when I feel very sleepy, I get easily distracted by other activities	BPS5	Impulse control/self‐regulation failure
	I do not go to bed on time	BPS6	Behavioral delay/poor routine adherence
	I have a regular bedtime routine	BPS7	Regularity/sleep hygiene
	I want to go to bed on time, but I just can't make myself do it	BPS8	Intention–action gap/self‐control failure
	When it's time to go to bed, I can easily stop what I am doing	BPS9	Self‐control/behavioral regulation

### 2.1 Within‐Person Temporal Network

**FIGURE 2:**
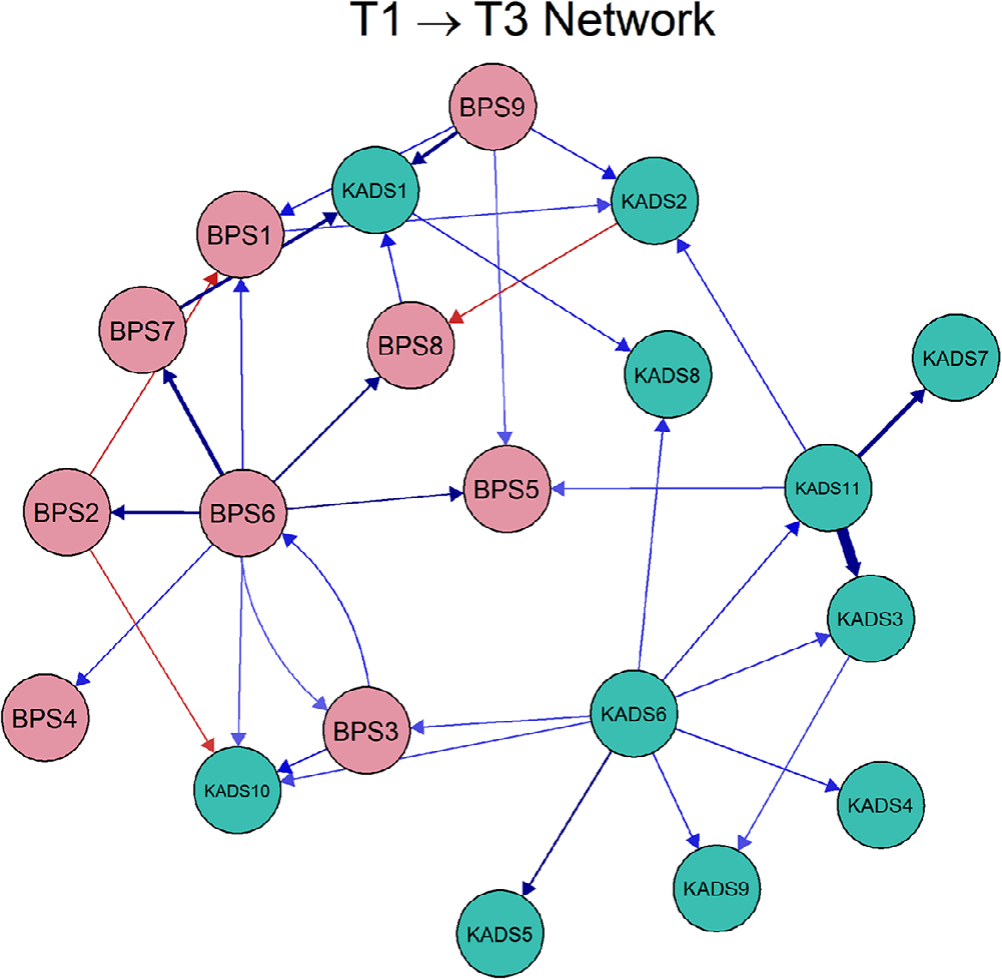
Cross‐lagged panel network of bedtime procrastination and depression for male adolescents, showing the cross‐lagged relationship between bedtime procrastination and depression.

**FIGURE 3:**
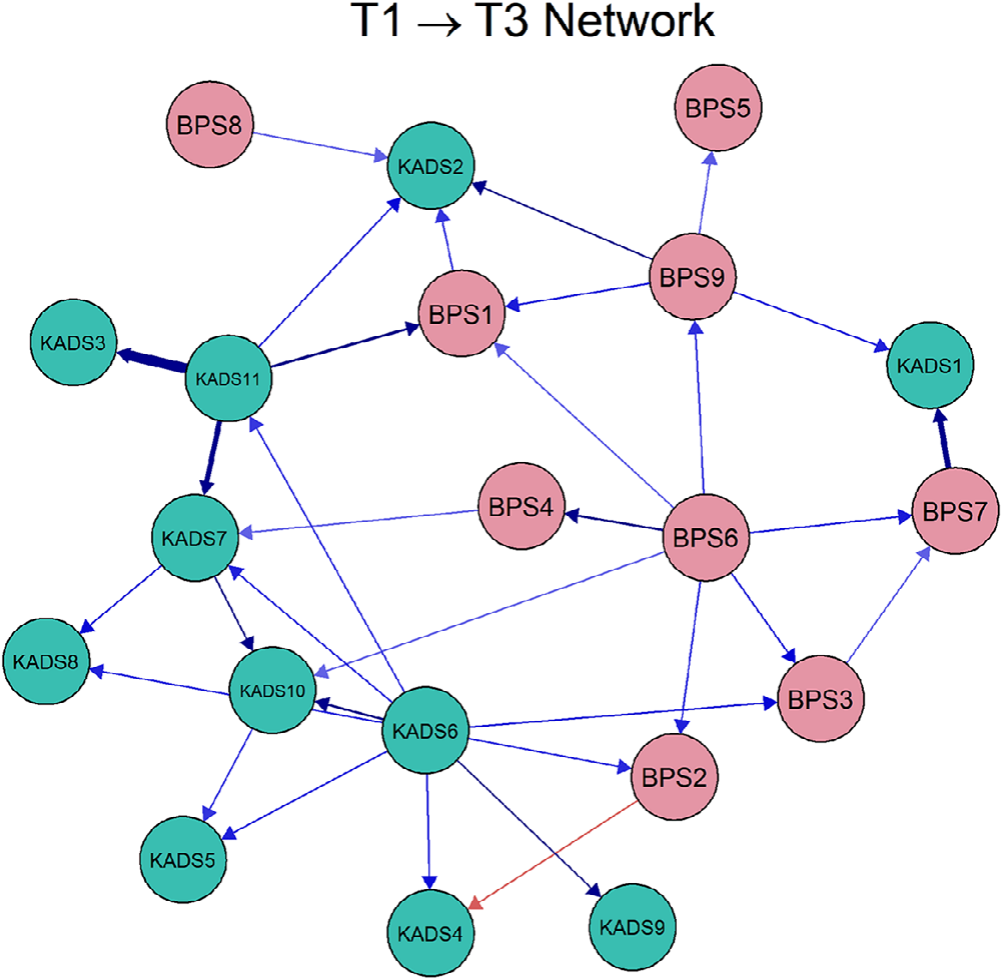
Cross‐lagged panel network of bedtime procrastination and depression for female adolescents, showing the cross‐lagged relationship between bedtime procrastination and depression.

Figures 2 and 3 illustrate the CLPN, displaying temporal networks with arrows indicating the time‐based associations between edges both within and across constructs. In the estimated network of males, the three strongest cross‐lagged edges were *Sleep difficulties* (KAD11) → *Feelings of worthlessness* (KAD3; *β* = 0.40), *Feelings of worthlessness* (KAD3) → *Feeling life is not very much fun* (KAD7; *β* = 0.37), as well as *Irregular bedtime* (BPS7) → *Physical feelings of worry* (KADS1; *β* = 0.32). Figure S1 presents the standardized node centrality estimates for male adolescents. The out‐EI centrality analysis showed that *not going to bed on time* (BPS6) had the highest OEI value (CS_OEI_ = 2.61) in the temporal network, indicating that this symptom was a strong longitudinal predictor of changes in both bedtime procrastination and depressive symptoms over time. In contrast, *physical feelings of worry* (KADS1) had the highest IEI value (CS_IEI_ = 2.21), indicating that they were significantly influenced by other symptoms within the network.

While in the estimated network for females, the three strongest cross‐lagged connections were *Sleep difficulties* (KAD11) →* Feelings of worthlessness* (KAD3; *β* = 0.45), *Irregular bedtime* (BPS7) → *Physical feelings of worry* (KADS1; *β* = 0.43), as well as *Sleep difficulties* (KAD11) → *Feeling life is not very much fun* (KADS7; *β* = 0.40). The standardized centrality parameters for the cross‐lagged panel networks in females are presented in Figure S2.* Trouble concentrating* (KADS6) showed a significantly higher out‐expected‐influence value (CS_OEI_ = 2.32) than other symptoms in the network. On the contrary, the symptom with the highest in‐expected‐influence value was *physical feelings of worry* (KADS1; CS_IEI_ = 2.18). Edge weight difference tests for all networks (both male and female adolescents) revealed that the aforementioned edges, excluding the exceptions, were significantly stronger (*p* < 0.05) compared to the majority of other edges (Figure S3). Temporal network metric coefficients showed strong stability for OEI estimations (CS_Male_ = 0.594; CS_Female_ = 0.672) and IEI (CS_Male_ = 0.517; CS_Female_ = 0.672). Additionally, the case‐drop bootstrapping analysis revealed moderate to high stability for both IEIs and OEIs, suggesting that these temporal network metrics are robust across different sample variations (see Figure S4).

### 2.2 Within‐Person Contemporaneous Network

Figures S5 and S6 display the contemporaneous networks that present the undirected edges within and across constructs for male and female adolescents. The stability of the centrality indices demonstrated that node strength in these two networks was estimated reliably, with strong strength coefficients (CS_Male_ = 0.750; CS_Female_ = 0.750) and expected influence coefficients (CS_Male_ = 0.750; CS_Female_ = 0.750). Across network clusters for males, the nodes exhibiting the highest strength centrality were *feeling worried* (KADS4) and *not going to bed on time* (BPS6). In contrast, for female adolescents, the central nodes with the highest strength centrality were *feeling tired* (KADS9) and *feeling worried* (KADS4). Additionally, the NCT was conducted to examine the differences in network structure and connection strength between male and female adolescents. No significant difference was found in the global network strength between sexes (*S* = 0.10, *p* = 0.75). Similarly, there were no statistically significant differences in the overall network structure estimates between male and female adolescents (*M* = 0.48, *p* = 0.09). However, several edges within the network were found to differ significantly. The three most significantly different edges in the two networks were observed between *trouble concentration* (KADS6) and *feeling life is not very much fun* (KADS7), *going to bed later than planned* (BPS1) and *doing things when should be sleeping* (BPS4), as well as *feeling life is not very much fun* (KADS7) and *sleep difficulties* (KAD11). To facilitate interpretation of the network analyses, we summarized the top central symptoms for males and females, respectively, in Table S1.

### 2.3 Between‐Person Network

**FIGURE 4:**
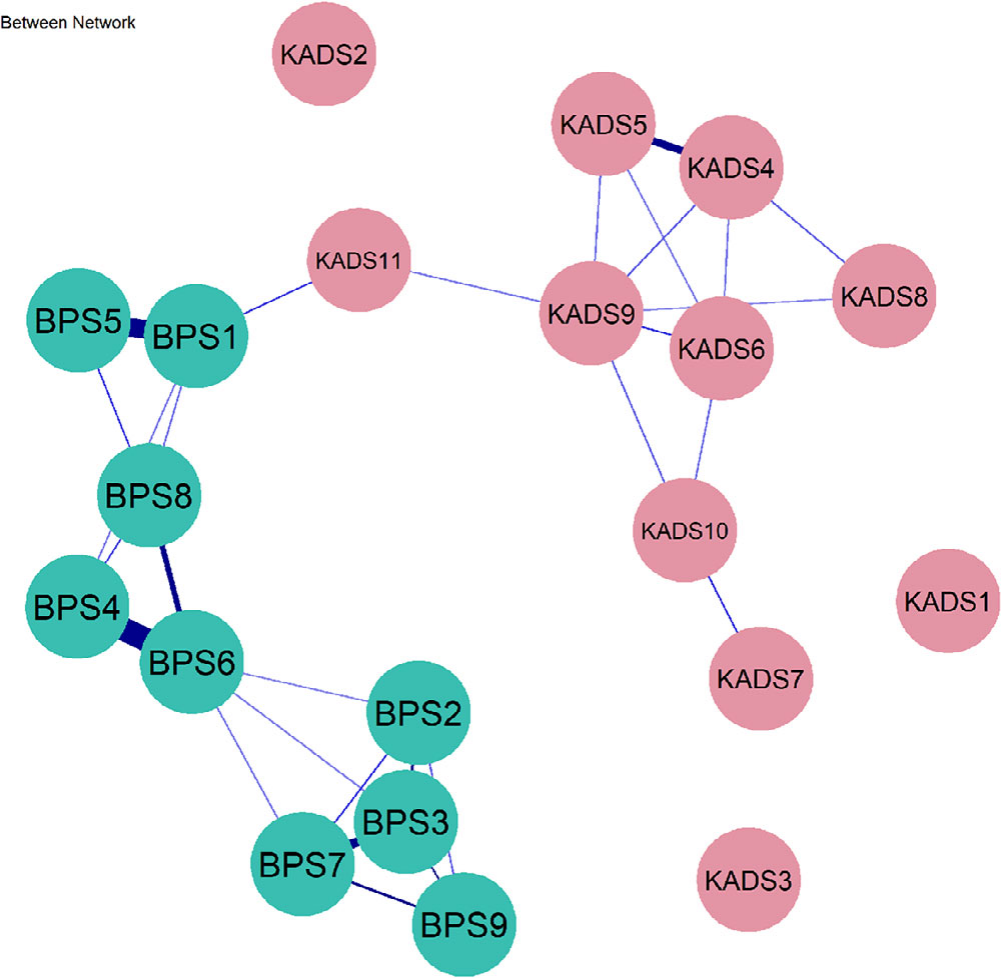
Between‐person network of bedtime procrastination and depression for male adolescents, illustrating the interconnections between bedtime procrastination and depression across individuals.

**FIGURE 5:**
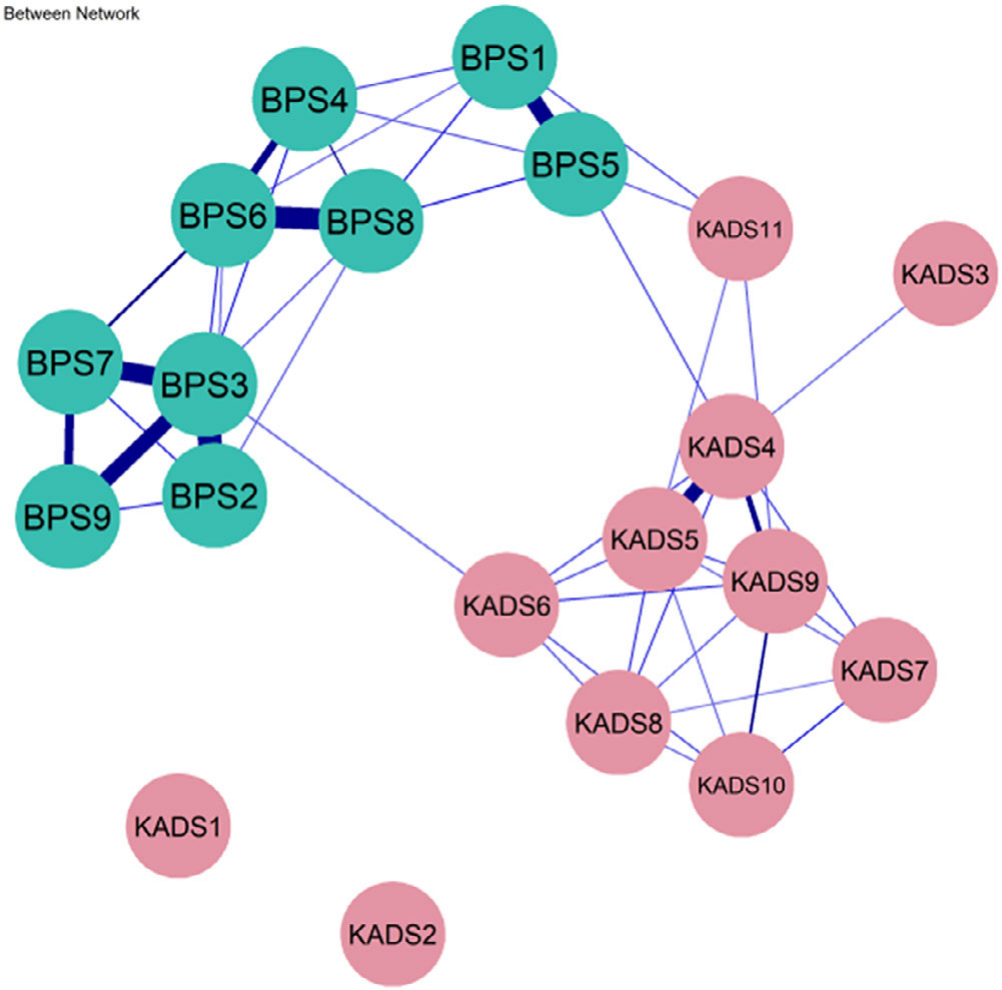
Between‐person network of bedtime procrastination and depression for female adolescents, illustrating the interconnections between bedtime procrastination and depression across individuals.

Figure 4 shows that for males, *feeling worried* (KADS4) is strongly associated with *irritability* (KADS5; *β* = 0.25). *Doing things when one should be sleeping* (BPS4) was also significantly related to *not going to bed on time* (BPS6, *β* = 0.33), while *going to bed later than planned* (BPS1) was likely to result in *getting distracted by other things even when sleepy* (BPS5, *β* = 0.31) between persons. For females, between persons, the symptom of *don't turn off the lights right away at bedtime* (BPS3) shows strong correlations with *don't go to bed early, even have to wake up early* (BPS2, *β* = 0.38), *irregular bedtime* (BPS7, *β* = 0.34), as well as *stopping things easily and sleep on time* (BPS9, *β* = 0.31). In addition, *not going to bed on time* (BPS6) is highly correlated with the symptom of *trying to go to bed on time but can't* (BPS8; *β* = 0.36). Details could be found in Figure 5.

## 3 Discussion

This study is the first to use a longitudinal network approach to characterize the dynamic symptom‐to‐symptom relationships between bedtime procrastination and depression in a large adolescent cohort, examining both within‐ and between‐person levels. By constructing distinct symptom networks for male and female adolescents, the findings reveal sex‐specific patterns in how symptoms are interrelated within and across individuals. These results provide valuable insights into the complex interconnections between bedtime procrastination and depression, highlighting the need for tailored interventions that target both behavioral and emotional dimensions in adolescent well‐being, with consideration of sex differences.

As for males, the three strongest cross‐lagged edges in the longitudinal within‐person network (temporal network) were identified: Sleep difficulties (KAD11) → Feelings of worthlessness (KAD3), Feelings of worthlessness (KAD3) → Feeling life is not very much fun (KAD7), and Irregular bedtime (BPS7) → Physical feelings of worry (KADS1). A previous study found that sadness, worthlessness, and insomnia are common depressive symptoms among adolescents [31]. Furthermore, irregular sleep patterns have been linked to worse physical and mental health conditions [32]. Our study also found that not going to bed on time (BPS6) affects other symptoms within the network, highlighting its central role in the relationship between bedtime procrastination and depression at the within‐person level. The presence of this symptom (BPS6) may indicate difficulties in self‐regulation, as bedtime procrastination is considered as a manifestation of poor self‐regulation [33]. In addition, the self‐regulatory dysfunction is a significant risk factor for depression [34, 35]. On the other hand, the depressive symptom of physical feelings of worry (KADS1) is most strongly predicted by other symptoms. This finding is in line with previous research suggesting that sleep insufficiency caused by bedtime procrastination and depression may precede the onset of certain chronic physical illnesses [36, 37].

Additionally, longitudinal within‐person network identified similar strongest cross‐lagged edges for female and male adolescents, except for the predictive relationships between sleep difficulties (KAD11) and feeling life is not very much fun (KAD7). We also found that depressive symptom of trouble concentrating (KADS6) significantly influences other symptoms at later time points in the female network. Previous research has shown that females tend to experience difficulties with concentration and suffer from nighttime sleep disturbances [38]. Additionally, studies have indicated that difficulty concentrating is associated with depression [39]. Similar to males, the physical feelings of worry (KADS1) were also the symptom most influenced by other symptoms.

The contemporaneous network reveals that feeling worried (KADS4) and not going to bed on time (BPS6) have high centrality in the within‐person network for male adolescents. It has been well documented that worry is closely related to depression and bedtime procrastination [40, 41]. Additionally, in female adolescents, the central nodes with the greatest strength centrality were feeling tired (KADS9) and feeling worried (KADS4). A cross‐sectional network analysis indicated that “feeling tired” was a core symptom of anxiety, depression, and academic burnout during late adolescence [42]. In addition, prior research has found that individuals who procrastinate on getting enough sleep tend to exhibit greater tiredness [43].

Notably, at the between‐person level, the positive relationships between certain items were stable. In the male network, feeling worried (KADS4) was significantly correlated with irritability (KADS5), consistent with previous findings [44]. Furthermore, engaging in activities when one should be sleeping (BPS4) was closely associated with not going to bed on time (BPS6). Meanwhile, adolescents who went to bed later than planned (BPS1) were also more likely to experience distractions despite feeling sleepy (BPS5). Previous findings have indicated that habitual behaviors, such as using social networking sites and smartphones, can disrupt university students’ ability to maintain adequate sleep duration [45, 46].

Our findings carry both theoretical and practical implications. While previous research has predominantly relied on cross‐sectional studies at the between‐person level in psychometric evaluation, network approaches enable the simultaneous analysis of multiple variables at the within‐person level. In this study, we extend existing work by providing new theoretical insights into the association between bedtime procrastination and depression, exploring differences across within‐person temporal, within‐person contemporaneous, and between‐person networks, with a particular attention to potential sex differences. From a practical perspective, our findings highlight the importance of designing interventions that target core symptoms and the most influential nodes within the network of bedtime procrastination and depression. Additionally, intervention strategies should account for both between‐person and within‐person changes in this interrelationship, as well as potential sex‐specific effects.

Several limitations should be acknowledged. First, the use of self‐reported questionnaires, including the KADS for assessing depressive symptoms, may have introduced response bias and may not fully capture the broader spectrum of symptoms. The adoption of more reliable measurements, such as multi‐informant reports from parents, teachers, or classmates, is recommended. Second, the study sample comprised general adolescents rather than those with psychiatric disorders. Future studies should aim to validate the results in clinical samples. Third, while our study examined both within‐ and between‐person networks, the current analytic approach does not allow for the formal testing of bridging effects across these levels simultaneously. As a result, we cannot determine whether certain symptoms, such as sleep difficulties, function as bridges linking bedtime procrastination and depressive symptoms across individuals. Finally, the network analysis did not account for external variables that may potentially influence the patterns of bedtime procrastination and depression, which warrants further exploration in future studies.

In summary, this study provides important insights into the dynamic relationships between bedtime procrastination and depression among adolescents, identifying key symptoms such as sleep difficulties, worry, fatigue, and concentration difficulties as critical targets for intervention. For male adolescents, interventions should target sleep hygiene and anxiety, while for females, focus should be on fatigue reduction and cognitive support. Additionally, between‐person effects indicate the importance of addressing persistent symptoms like irregular sleep, which may exacerbate depressive symptoms. This highlights the importance of developing personalized interventions that address both within‐person symptom dynamics and between‐person symptom profiles. School‐based screening and digital monitoring can facilitate the early identification of adolescents with high‐impact symptoms, enabling timely and personalized interventions. By incorporating both within‐person dynamics and between‐person differences, interventions can be more effective and responsive to individual needs. A sex‐sensitive approach that targets specific symptom patterns can help reduce the long‐term burden of both bedtime procrastination and depression in adolescents.

## 4 Methods

### 4.1 Study Design and Participants

Data were collected from students in Grades 7–12 at two junior high schools and one senior high school located in Shandong Province, China, over a period of 1.5 years. The study was initiated in November 2021 with the first measurement (T1), followed by the second survey 6 months later in May 2022 (T2). The third (T3) data collection, conducted at a 1‐year interval, was held in May 2023. The unequal time intervals were designed to capture both short‐term and longer term symptom fluctuations. The present study employed a two‐wave longitudinal design, utilizing data from Time 1 and follow‐up questionnaires conducted 1.5 years later (T3) to examine the long‐term developmental trajectories of bedtime procrastination and depressive symptoms over an 18‐month period, reducing model complexity and focusing on more stable and representative interrelations between constructs.

Participants were recruited through school coordination. Inclusion criteria required full‐time enrollment and the ability to complete questionnaires independently, while students with severe mental or developmental disorders were excluded. Prior to data collection, written informed consent was obtained from all participants, including the guardians of minors. All participants were informed of the study's objectives before participation and assured of the anonymity and confidentiality of their research data. Participants completed paper‐based questionnaires to evaluate their behavioral and mental health status, which were administered in designated classrooms under the guidance of trained research staff. This study was conducted in accordance with the Declaration of Helsinki and was approved by the Research Ethics Committee of the School of Public Health, Shandong University.

### 4.2 Measures

#### 4.2.1 Bedtime Procrastination

Bedtime procrastination behaviors were assessed using the Bedtime Procrastination Scale (BPS) [47]. It comprised nine items with a five‐point Likert response, ranging from 1 (almost never) to 5 (almost always). In the present study, item‐level data from all nine BPS items were used for network estimation, as each item was treated as a distinct node reflecting a specific aspect of procrastination behavior. The BPS exhibited robust reliability and validity in a sample of Chinese university students [48]. Cronbach's alpha was 0.83 at T1 and 0.85 at T3 in this study.

#### 4.2.2 Depression

Depression was measured by the Kutcher Adolescent Depression Scale (KADS), which is a self‐reported measure consisting of 11 items specifically used to assess depressive symptoms among adolescents [49]. It was initially validated in a Canadian population and has been adapted into a Chinese version, demonstrating proven reliability and validity [50]. The scale assesses core depressive symptoms such as low mood, sleep disturbances, and suicidal ideation/self‐harm. Participants were asked to assess the frequency of these symptoms experienced over the past week, using a 4‐point scale ranging from 0 (*hardly ever*) to 3 (*all of the time*). Consistent with the analytic focus of this study, each KADS item was modeled as an individual node within the symptom network, capturing fine‐grained dynamics of depressive experiences. The Cronbach's α for KADS‐11 was 0.92 at T1 and 0.93 at T3.

### 4.3 Statistical Analyses

The cross‐sectional and cross‐lagged network analyses were conducted to estimate the network dynamics using R software (v.4.1.1). This approach utilizes a Gaussian graphical model (GGM) with graphical LASSO (Least Absolute Shrinkage and Selection Operator) combined with an extended Bayesian information criterion (EBIC) for model selection. This method regularizes the magnitude of each regression weight, effectively setting small coefficients to zero [51]. Each symptom item (nine from the BPS and eleven from the KADS) was represented as a node. While edges between nodes reflected partial correlations (for cross‐sectional networks) or directed temporal effects (for longitudinal networks). Edge thickness represented the magnitude of association; blue lines indicated positive associations, and red lines represented negative ones [20]. In addition, we performed permutation‐based tests of network invariance (M) and global strength invariance (S) to assess overall network differences using the NetworkComparisonTest (NCT) function [52].

To clarify the interrelationships between baseline and follow‐up assessments over time, CLPN was employed [53]. This method demonstrates how individual items at one time point predict other nodes at the subsequent time point, by evaluating both cross‐lagged and autoregressive effects. Autoregressive paths emerged as the strongest in the network, which suppressed the cross‐lagged paths. Consequently, to highlight the cross‐lagged effects most relevant to the study's goals, the autoregressive paths were set to zero [53]. Centrality indices, such as in‐expected influence (IEI) and out‐expected influence (OEI), were utilized to evaluate the centrality of symptoms within the CLPNs. IEI quantifies the extent to which a symptom is influenced by other symptoms, whereas OEI indicates the extent to which a symptom is predicted by other symptoms at subsequent time points. To further separate temporal inferences between and within persons, we applied the panel graphical vector autoregressive (panelgvar) model [54] to construct a between‐person network. This model highlights the partial associations reflecting stable trait‐level differences over time. The model was fit using a non‐regularized approach as described by Speyer et al. [55].

The R package “bootnet” was applied to check edge and centrality stability using bootstrapping [56]. The non‐parametric bootstrap method was employed to assess the accuracy of edge weights, generating 95% confidence intervals (CIs) by resampling the data with replacement. Additionally, the case‐drop bootstrap method was applied to assess the stability of centrality indices by comparing correlations of centrality estimates between the original and subset samples. Stability was confirmed if indices showed minimal change in samples, excluding up to 70% of the original data. Correlation stability (CS) coefficients were computed, with a recommended threshold of 0.50 or higher, to ensure the reliability and stability of the network measures across resampling.

The amount of missing data was minimal, accounting for less than 10% of missing values across all study variables. Little's MCAR test suggested that data were missing at random (*p* > 0.05). Therefore, multiple imputation was employed by the multivariate imputation using the chained equations (MICE) method available in R (version 4.1.1). Ten imputed datasets were generated using predictive mean matching for continuous variables and logistic regression for categorical variables. Parameter estimates from subsequent analyses were pooled according to Rubin's rules to account for imputation uncertainty.

## Author Contributions

T.G. conceived of the study, participated in its design and coordination, and drafted the manuscript. W.Z. participated in the design and interpretation of the data. Y.S. supervised the study, revised the manuscript critically for important intellectual content, and approved the final version. All authors read and approved the final manuscript.

## Funding

This research was supported by the National Natural Science Foundation of China (72204144), Humanities and Social Sciences Youth Fund of the Ministry of Education of China (21YJC630028 and 24YJCZH258), Natural Science Foundation of Shandong Province (ZR2021QG052), Shandong Social Science Planning Fund Program (21CSZJ31), Shenzhen Science and Technology Innovation Program (JCYJ20240813094517023), and the Fund of the Science and Technology Planning Project of Shenzhen Municipality (20210617155253001).

## Ethics Statement

Written informed consent was obtained from all participants and the guardians of minors. Ethics approval (LL20210102) was obtained from the Research Ethics Committee of the School of Public Health, Shandong University, prior to the commencement of participant recruitment.

## Conflicts of Interest

The authors declare no conflicts of interest.

## Supporting information




**Table S1**: Comparison of network centrality and structure between male and female adolescents in within‐person temporal and contemporaneous networks.
**Figure S1**: Centrality estimation for male adolescents.
**Figure S2**: Centrality estimation for female adolescents.
**Figure S3**: Edge weight difference tests for the networks using T1 to predict T3 among male and female adolescents. Black boxes indicate edges that significantly differ (p < 0.05), and gray boxes indicate edges that do not significantly differ.
**Figure S4**: Stability of the centrality indices in the CLPN for male and female adolescents.
**Figures S5**: The cross‐sectional network of bedtime procrastination and depression for male adolescents.
**Figures S6**: The cross‐sectional network of bedtime procrastination and depression for female adolescents.

## Data Availability

The minimum anonymized data that support the findings of this study are available upon reasonable request from the corresponding authors.
