# Supplementary material for: Temporal Network Analysis of Bedtime Procrastination and Depression Among Adolescents: A Prospective Longitudinal Study
Source: MedComm (2020). 2026 Jan 25;7(2):e70620. doi: 10.1002/mco2.70620 (PMC12832071; doi:10.1002/mco2.70620)
Supplement: Supplementary file 1 — Table S1: Comparison of network centrality and structure between male and female adolescents in within‐person temporal and contemporaneous networks. Figure S1: Centrality estimation for male adolescents. Figure S2: Centrality estimation for female adolescents. Figure S3: Edge weight difference tests for the networks using T1 to predict T3 among male and female adolescents. Black boxes indicate edges that significantly differ (p < 0.05), and gray boxes indicate edges that do not significantly differ. Figure S4: Stability of the centrality indices in the CLPN for male and female adolescents. Figures S5: The cross‐sectional network of bedtime procrastination and depression for male adolescents. Figures S6: The cross‐sectional network of bedtime procrastination and depression for female adolescents. [file MCO2-7-e70620-s001.docx]

**Temporal network analysis of bedtime procrastination and depression among adolescents: A prospective longitudinal study**

Tingting Gao^1,2,3,4^, Wei Zhang^1,2,3^, Yingying Su^5*^

^1^ Department of Social Medicine and Health Management, School of Public Health, Cheeloo College of Medicine, Shandong University, Jinan, Shandong, China

^2^ NHC Key Lab of Health Economics and Policy Research (Shandong University)

^3^ Center for Health Management and Policy Research, Shandong University (Shandong Provincial Key New Think Tank)

^4^ School of Public Health, Wannan Medical College, Wuhu, Anhui, China

^5^ School of Public Health and Emergency Management, Southern University of Science and Technology, Shenzhen, Guangdong, China

**^*^Corresponding author:**

^*^ Yingying Su, School of Public Health and Emergency Management, Southern University of Science and Technology, 1088 Xueyuan Avenue, Shenzhen, Guangdong, 518055, China (suyy3@sustech.edu.cn);

**Telephone:** + 86-15219468706

Table S1. Comparison of network centrality and structure between male and female adolescents in within-person temporal and contemporaneous networks.

| Within-person temporal network | | |
| --- | --- | --- |
| Symptoms | Male adolescents | Female adolescents |
| Out-expected-influence (OEI) | *Don’t go to bed on time* (BPS6) | *Trouble concentrating* (KADS6) |
| In-expected-influence (IEI) | *Physical feelings of worry* (KADS1) | *Physical feelings of worry* (KADS1) |
| Within-person contemporaneous network | | |
| Strength centrality | *Feeling worried* (KADS4)  *Don’t go to bed on time* (BPS6) | *Feeling tired* (KADS9)  *Feeling worried* (KADS4) |


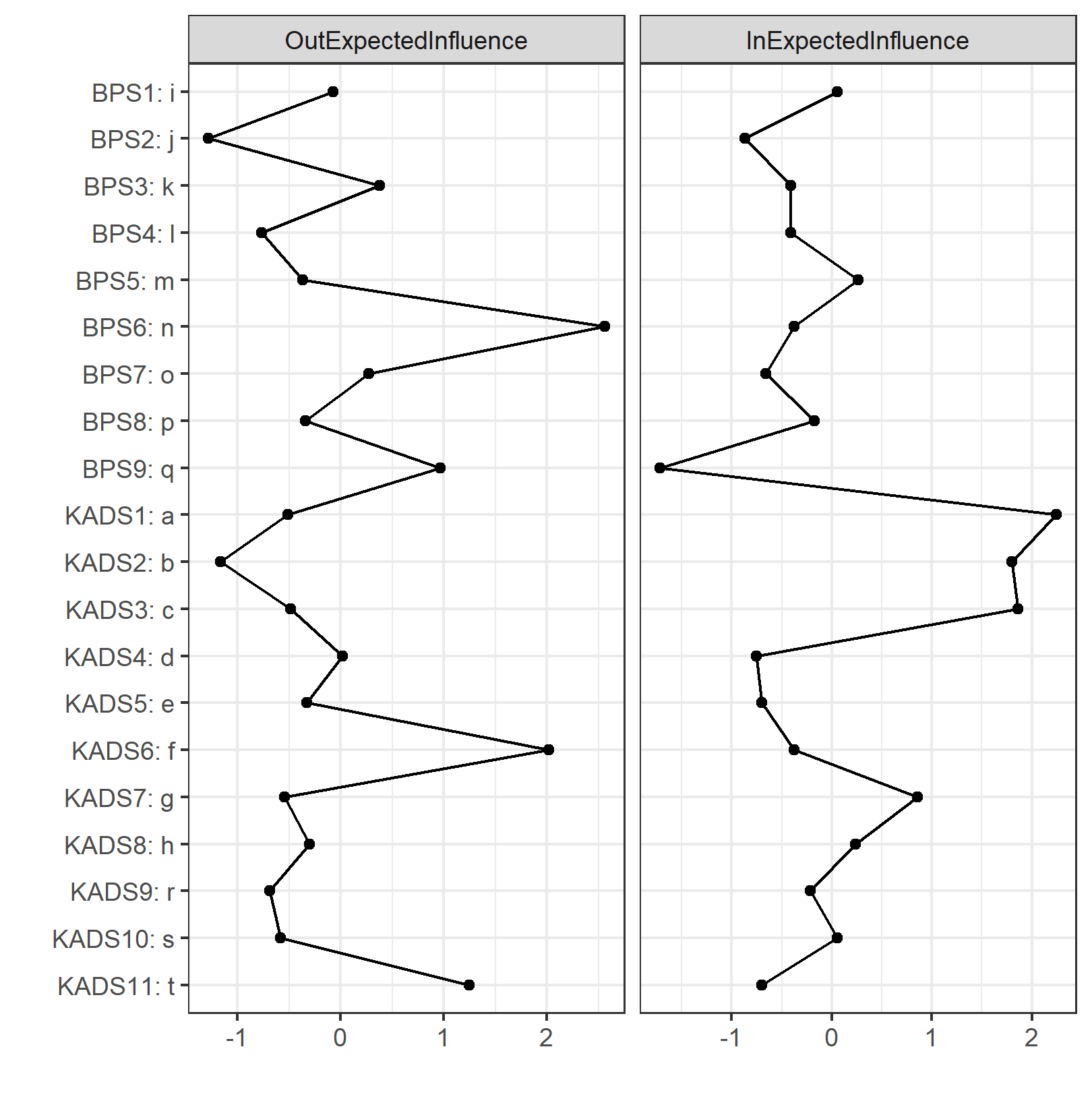
Figure S1. Centrality estimation for male adolescents.


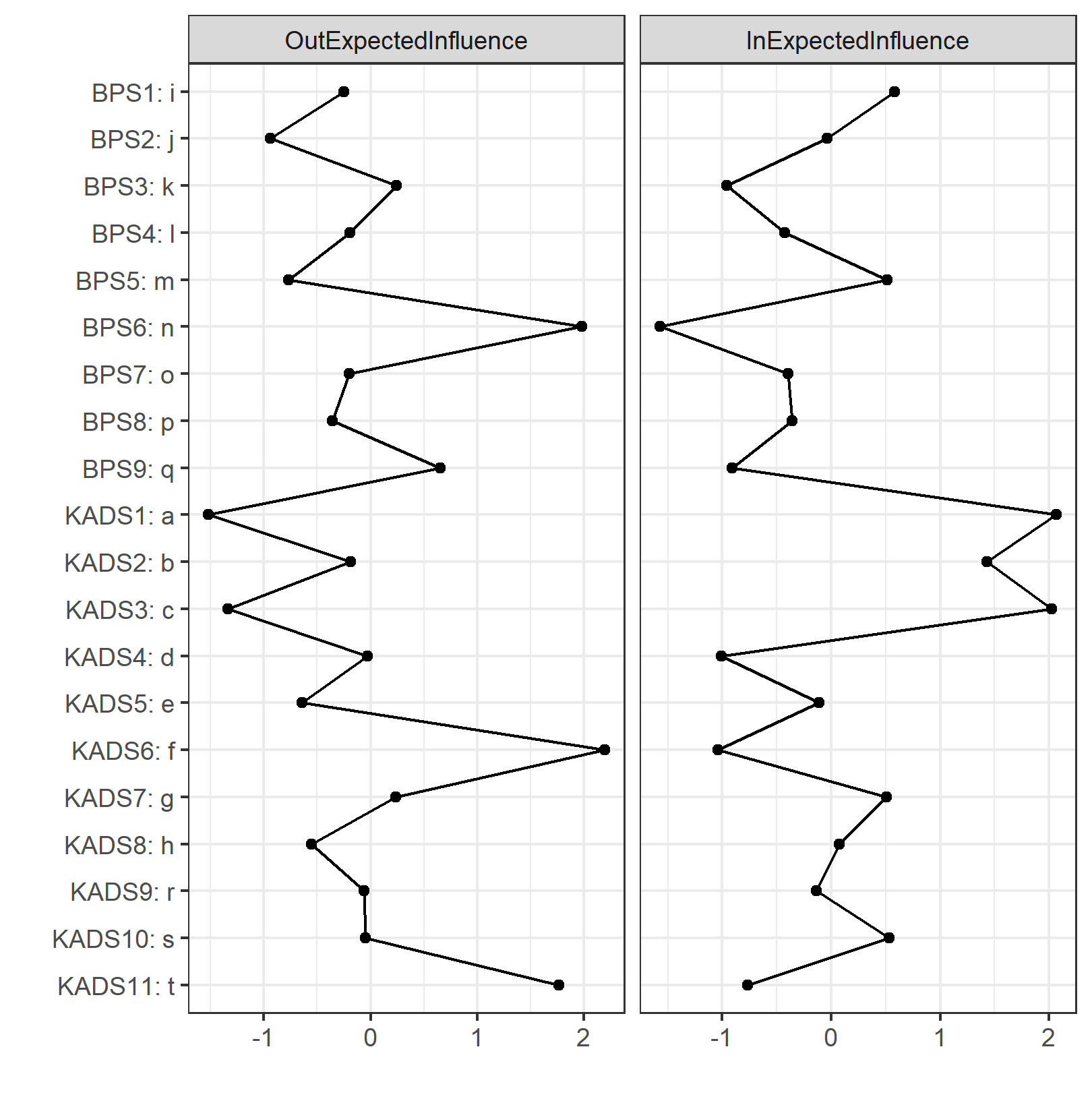


Figure S2. Centrality estimation for female adolescents.


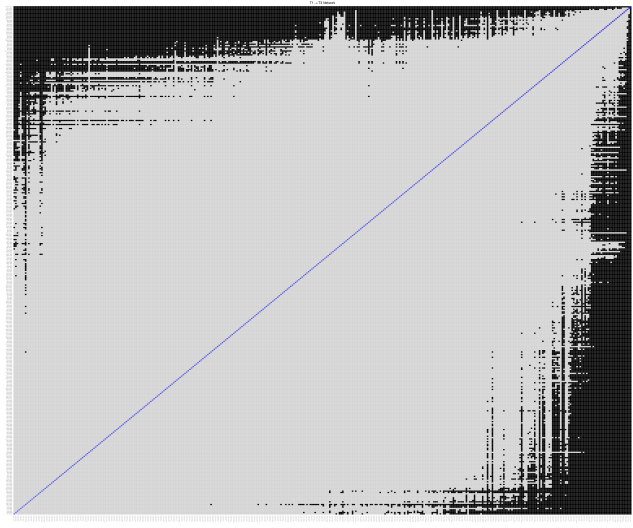

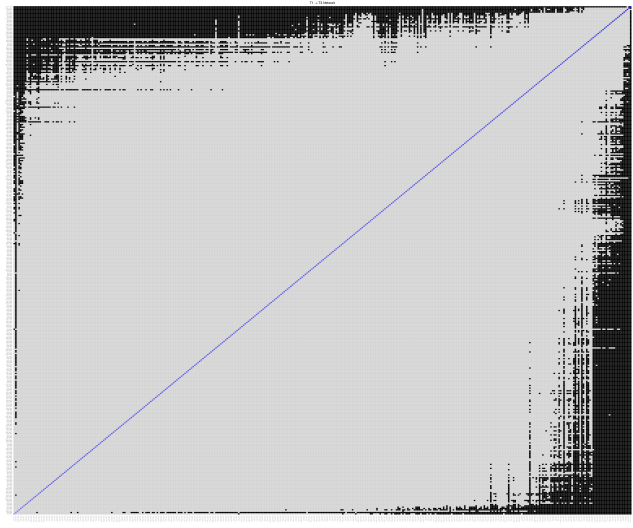


1. Male (B) Female

Figure S3. Edge weight difference tests for the networks using T1 to predict T3 among male and female adolescents. Black boxes indicate edges that significantly differ (p < 0.05), and gray boxes indicate edges that do not significantly differ.


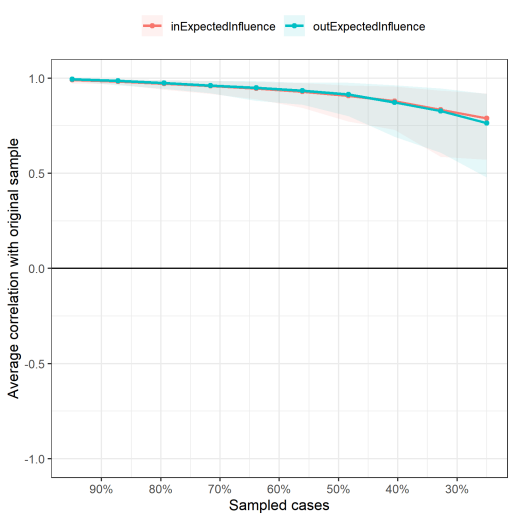

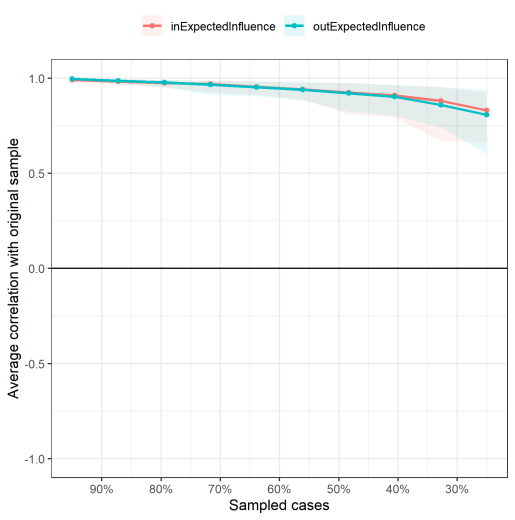


(A) Male (B) Female

Figure S4. Stability of the centrality indices in the CLPN for male and female adolescents.


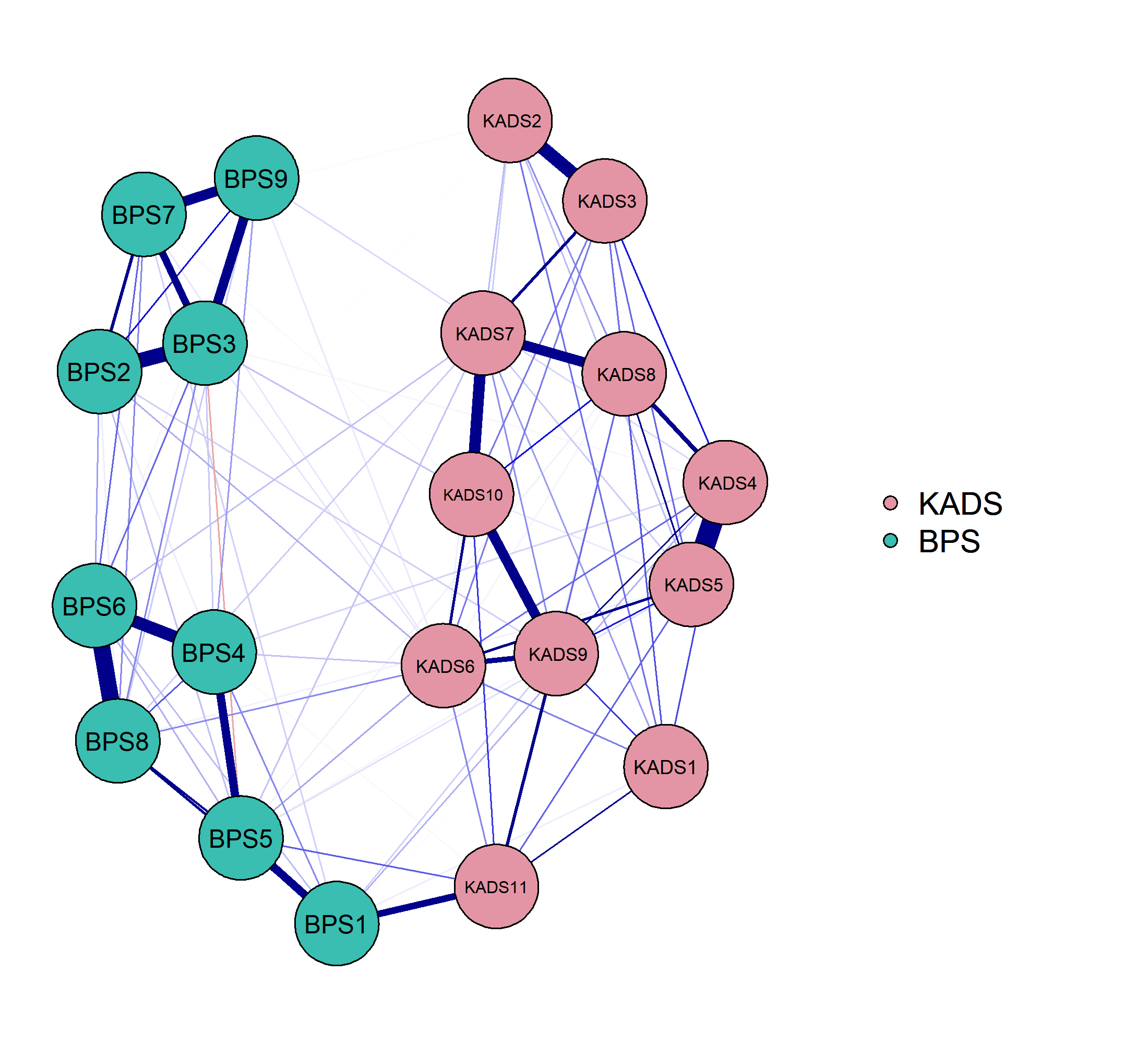


Figures S5. The cross-sectional network of bedtime procrastination and depression for male adolescents.


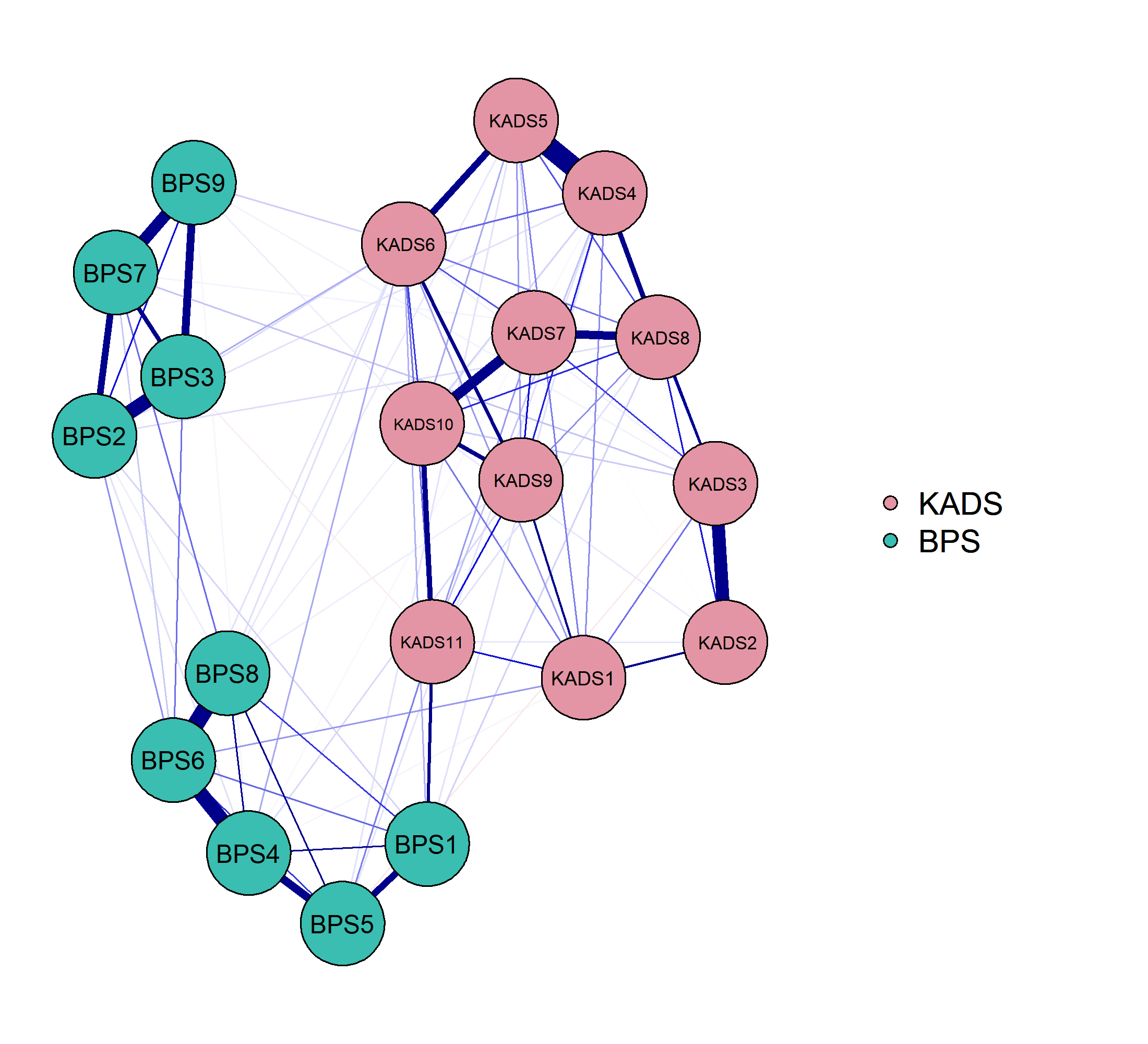


Figures S6. The cross-sectional network of bedtime procrastination and depression for female adolescents.
